# Severe Multifocal Cutaneous Sporotrichosis Caused by *Sporothrix brasiliensis* in People Living with HIV in Southern Brazil: Cases and Review

**DOI:** 10.3390/pathogens15101056

**Published:** 2026-10-06

**Authors:** Vanice Rodrigues Poester, Bianca dos Santos Blan, Rossana Patricia Basso, Mariana Rodrigues Trápaga, Lívia Silveira Munhoz, Monica Campos dos Santos, Karine Ortiz Sanchotene, David A. Stevens, Melissa Orzechowski Xavier

**Affiliations:** 1 Programa de Pós-Graduação em Ciências da Saúde, Faculdade de Medicina (FAMED), Universidade Federal do Rio Grande (FURG), Rio Grande 96200-190, RS, Brazil; vanicerp@gmail.com (V.R.P.); biancablanenf@gmail.com (B.d.S.B.); marianartrapaga@gmail.com (M.R.T.); liviasmunhoz@gmail.com (L.S.M.); monicacampos978@gmail.com (M.C.d.S.);; 2 Laboratório de Micologia, FAMED, FURG, Rio Grande 96200-190, RS, Brazil; rossanabasso3@gmail.com (R.P.B.); kasanchotene@gmail.com (K.O.S.); 3 Hospital Universitário Dr. Miguel Riet Corrêa Jr., FURG, Empresa Brasileira de Serviços Hospitalares (Ebserh) (HU-FURG/Ebserh), Rio Grande 96200-190, RS, Brazil; 4 Division of Infectious Diseases and Geographic Medicine, Stanford University Medical School, Stanford, CA 94305, USA

**Keywords:** sporotrichosis, *Sporothrix*, endemic fungi, zoonosis, AIDS, immunosuppression, hyperendemic area

## Abstract

Background: *Sporothrix brasiliensis*-associated sporotrichosis is an emerging zoonotic mycosis in South America, especially in Brazil. Whereas sporotrichosis is classically described as a lymphangitic disease, multifocal forms occur, predominantly in people living with HIV (PLHIV). We report two cases of severe multifocal cutaneous sporotrichosis caused by *S. brasiliensis* in PLHIV from a hyperendemic region in southern Rio Grande do Sul, Brazil, and review the relevant literature. Methods and Results: Both patients presented advanced immunosuppression, low CD4+ T-cell counts, and poor adherence to antiretroviral therapy, which contributed to disease progression. The first patient developed recurrent multifocal lesions over a two-year period, whereas the second progressed from extensive mucocutaneous involvement to multifocal disease with partial destruction of nasal cartilage, resulting in permanent sequelae. In both cases, *S. brasiliensis* was confirmed by culture and species-specific polymerase chain reaction. Isolates from both cases exhibited wild-type susceptibility to itraconazole. Treatment with itraconazole was given, with no susceptibility changes in sequential isolates recovered during disease progression nor after antifungal therapy abandonment. Conclusion: These cases highlight that extensive tissue destruction and permanent deformities, resembling that from other systemic mycoses, can occur in PLHIV. Our findings and review reinforces the importance of early HIV investigation, adherence to antiretroviral and antifungal therapies, and continuous epidemiological and antifungal susceptibility surveillance in hyperendemic regions.

## 1. Introduction

*Sporothrix brasiliensis* is an emergent fungal pathogen in South America impacting the public health system. In Brazil, sporotrichosis was recently added to the national list of mandatory notifiable disease. There are more than 8000 human cases and more than 7000 feline cases reported in the scientific literature through 2023 [1]. Its zoonotic acquisition, through infected cats, represents the main route of dissemination [1,2]. In recent years, imported cases in the UK and the United States were reported, reinforcing its recognition as an emerging global health concern [1,3]. Concomitant with the emergence of *S. brasiliensis*-associated sporotrichosis, there has been an increase in reports of severe and/or extracutaneous clinical forms, including mucosal (particularly ocular and nasal), osteoarthritis, hypersensitivity reactions, meningeal, pulmonary, and multifocal manifestations (cutaneous or extracutaneous) [4,5,6,7,8,9].

Different prognostic profiles are observed among the clinical manifestations of sporotrichosis [8,10]. Although the incubation period of sporotrichosis is generally described as ranging from one to four weeks [11], cases in which disease manifestations occurred months or even years after exposure to the fungus have also been reported [12,13]. Cutaneous (fixed or lymphocutaneous), mucosal involvement, and hypersensitivity reactions are generally associated with a better prognosis and patients without severe immunosuppression [4,7,8,9,14,15,16]. In contrast, osteoarticular, meningeal, pulmonary, and multifocal (cutaneous or extracutaneous) forms are associated with a poorer prognosis, frequently requiring hospitalization and intravenous antifungal therapy, and are mainly observed in immunosuppressed patients, particularly people living with HIV (PLHIV) [5,6,8,10,15]. A systematic review of atypical sporotrichosis cases associated with *S. brasiliensis* identified 791 published cases over a 26-year period (1998–2023) across 11 Brazilian states [8]. Hypersensitivity reactions were the most frequently reported atypical presentation (47%; *n* = 370); mucosal (32%; *n* = 256), multifocal (8%; *n* = 60), osteoarticular (7%; *n* = 59), meningeal (4%; *n* = 32), and pulmonary (2%; *n* = 14) infections were less common. Among cases with available outcome data (*n* = 607), death occurred in 7% (*n* = 43) [8]. These findings highlight the broad clinical spectrum of atypical sporotrichosis caused by *S. brasiliensis* and underscore the importance of considering diverse clinical presentations, particularly in patients with underlying conditions or immunosuppression.

Focusing specifically on PLHIV, a systematic review including 25 studies published between 2015 and 2022 and comprising 52 PLHIV patients with sporotrichosis, multifocal extracutaneous disease was the most frequent clinical presentation (40/51), followed by multifocal cutaneous (8/51), together accounting for 94% of cases [17]. In contrast, the classical fixed cutaneous (2/51) and lymphocutaneous (1/51) forms were less frequently reported, accounting for approximately 6% of cases [17]. The mean CD4+ cell count among these patients was 104 cells/mm^3^, showing the association between advanced immunosuppression and the development of severe and multifocal forms of sporotrichosis in PLHIV. This association is further supported by the high mortality observed, with 42% of patients dying during follow-up [17]. Most cases were reported in Brazil (84%), suggesting a predominance of *S. brasiliensis* as the etiological agent in this population [17].

Regarding sporotrichosis treatment, itraconazole is the first-line therapy for fixed cutaneous and lymphocutaneous forms in humans, generally administered at 100 to 400 mg/day [1,3]. Terbinafine may be considered when itraconazole is contraindicated or unsuitable, such as during pregnancy or after bariatric surgery [3]. For multifocal forms and extracutaneous disease with systemic involvement, liposomal amphotericin B or amphotericin B deoxycholate is indicated at 3–5 mg/kg/day or 0.7–1 mg/kg/day, respectively, followed by oral itraconazole once clinical improvement is achieved [1,3]. Although amphotericin B deoxycholate is no longer the preferred formulation because of its higher toxicity, it remains as a therapeutic option in Brazil due to its availability through the Brazilian Unified Health System [3,10].

Southern Rio Grande do Sul (RS), Brazil, has been a hyperendemic area for *S. brasiliensis*-associated sporotrichosis since the 1990s [18]. Most reported cases over the years have presented as cutaneous forms, whereas extracutaneous manifestations, predominantly ocular involvement and hypersensitivity reactions, have only been described more recently [9,14,19]. One of the epicenters of this hyperendemic region is the city of Rio Grande, which ranks among the top 25% of Brazilian municipalities with more than 100,000 inhabitants according to the composite index of HIV cases [20]. Interestingly, despite the high incidence of both HIV infection and sporotrichosis in this city, only one multifocal sporotrichosis case in PLHIV, associated with immune reconstitution inflammatory syndrome (IRIS), has been previously reported [21]. Thus, the present study reports two other cases of multifocal cutaneous sporotrichosis caused by *S. brasiliensis* in PLHIV in southern RS, Brazil.

## 2. Materials and Methods

For the reported cases, clinical and epidemiological data were obtained from the Specialized Reference Service (SRS) for sporotrichosis of the University Hospital of the Federal University of Rio Grande/*Empresa Brasileira de Serviços Hospitalares* (HU-FURG/Ebserh), in Rio Grande, RS, Brazil [22], in association with data from the Mycology Laboratory of the FURG School of Medicine, Rio Grande, RS, Brazil. In this laboratory, diagnosis was performed by direct mycological examination and mycological culture, followed by species identification and antifungal susceptibility testing.

Mycological culture was performed on Sabouraud agar with and without cycloheximide and antibacterial agents, and direct mycological examination was performed using Grocott–Gomori staining. Samples were inoculated onto the culture media and incubated at 30 °C for up to 30 days or until fungal growth was observed. *Sporothrix* spp. were identified based on macro- and microscopic characteristics, including white to cream-colored colonies that darkened over time, with a membranous texture, moderate growth rate, and a star-shaped margin. Microscopically, slender, septate hyphae with characteristic conidia arranged singly or in daisy-like clusters were observed. Species identification was performed using a species-specific polymerase chain reaction (PCR) for *S. brasiliensis* with the primers SbraF (5′-CCCCCGTTTGACGCTTGG) and SbraR (5′-CCCGGATAACCGTGTGTCATAAT) and the reaction mixture and amplification conditions described in the protocol by Rodrigues et al. [23]. PCR products were analyzed by 1.5% agarose gel electrophoresis, and samples showing a 469-bp amplicon were considered positive for *S. brasiliensis* [23].

Isolates were evaluated for *in vitro* susceptibility to itraconazole (Sporanox^®^, Janssen-Cilag Farmacêutica Ltd., São Paulo, São Paulo, Brazil) and amphotericin B deoxycholate (Cristália, Itapira, São Paulo, Brazil), commercially obtained, using the Clinical and Laboratory Standards Institute (CLSI) protocol [24]. Briefly, isolates were standardized to a final inoculum concentration ranging from 0.8 × 10^4^ to 1 × 10^5^ colony-forming units/mL. Aliquots of 100 µL of the standardized inoculum were dispensed into microplate wells containing 100 µL of each antifungal agent [24]. Itraconazole and amphotericin B were tested at concentrations ranging from 0.0313 to 16 μg/mL. Isolates were classified as wild-type or non-wild-type according to the epidemiological cutoff value, with a cutoff of ≥8 µg/mL for both amphotericin B and itraconazole, following the CLSI definition [25].

In addition to our data, we performed a focused systematic review of the literature on multifocal cutaneous sporotrichosis caused by *Sporothrix brasiliensis*, which was incorporated into the Discussion section. For this purpose, we searched the PubMed database using the descriptors “multifocal cutaneous sporotrichosis” and “*Sporothrix brasiliensis*”.

## 3. Results

### 3.1. Case 1

**Figure 1:**
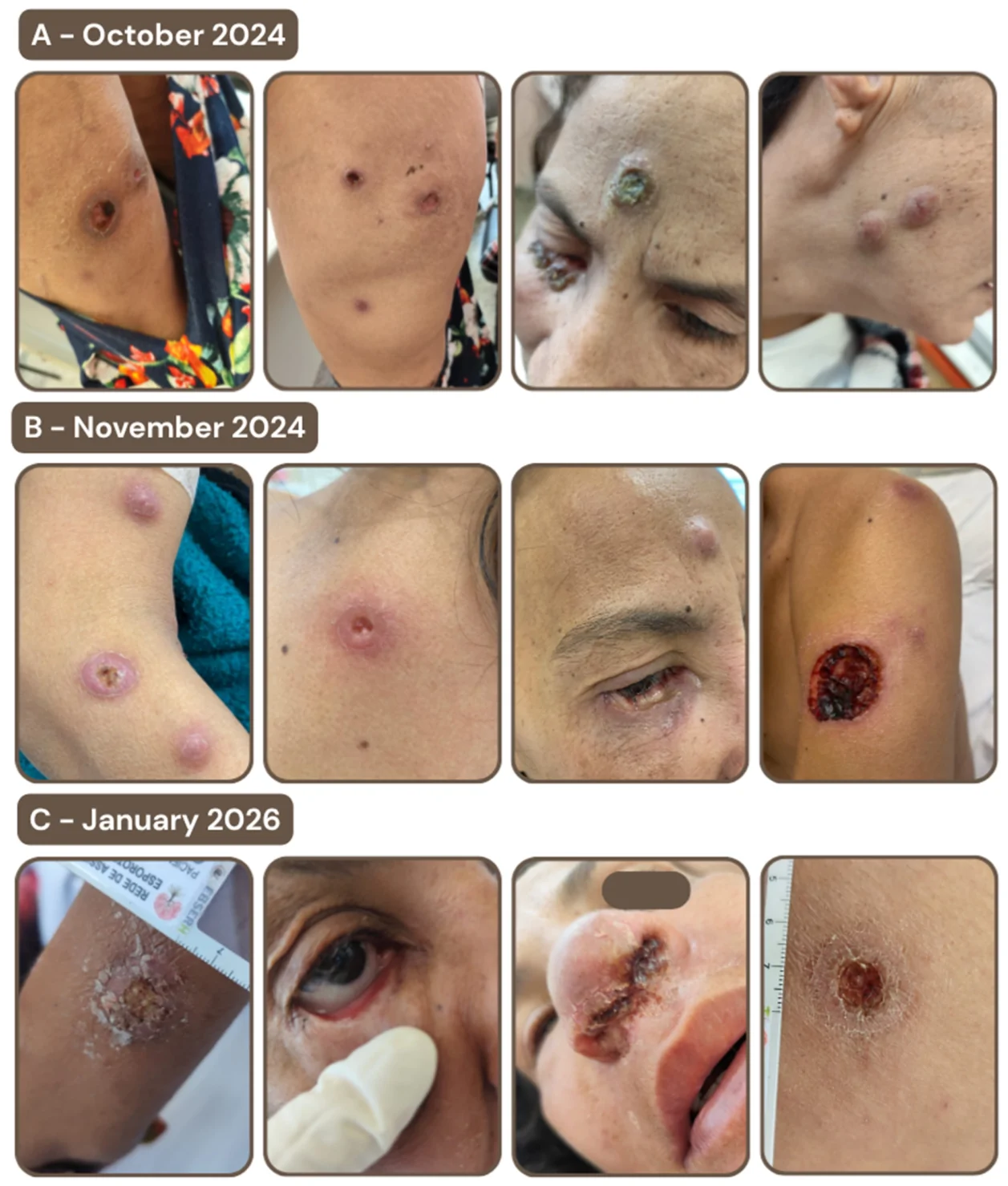
A 45-year-old woman living with HIV for 14 years, who was not receiving regular antiretroviral therapy, developed multifocal cutaneous sporotrichosis following contact with a stray cat. The patient had approximately five months of disease progression before diagnosis. (**A**) Initial clinical presentation of sporotrichosis caused by *Sporothrix brasiliensis* in October 2024. (**B**) Clinical progression after one month of follow-up (November 2024), following abandonment of antiretroviral therapy and itraconazole treatment (200 mg/day). (**C**) Follow-up evaluation in January 2026, showing progression of the cutaneous lesions and a new isolation of *S. brasiliensis*, following a second episode of abandonment of antiretroviral therapy and itraconazole treatment (400 mg/day).

In October 2024, a 45-year-old woman who self-identified as White with a history of drug addiction, social vulnerability, and HIV infection for 14 years, without using regular antiretroviral treatment, presented to the SRS/HU-FURG/Ebserh in Rio Grande, RS, Brazil [22]. The patient presented with right eyelid edema, conjunctival hyperemia associated with purulent discharge, and painful, erythematous, raised lesions involving the shoulders, elbows, and bilateral gluteal regions. The lesions were warm to the touch, ulcerated (8–10 cm in diameter), and exhibited raised, inflamed borders (Figure 1A). At this time, the patient’s HIV viral load was 74,700 copies/mL and the CD4+ T-cell count 131 cells/mm^3^. She reported that approximately five months before the onset of lesions, a stray cat had entered her home and remained on the sofa and bed where she slept. However, she denied any history of scratches, bites, or direct contact with cats.

Treatment with oral itraconazole (200 mg/day; Geolab^®^, Anápolis, Goiás, Brazil) was initiated, and oral antiretroviral therapy (ART) was reintroduced using tenofovir disoproxil fumarate (TDF; 300 mg/day), lamivudine (3TC; 300 mg/day), and dolutegravir (DTG; 50 mg/day). Biopsy specimens from lesions on the upper limb and buttock were collected for mycological analysis. The culture confirmed the presence of *Sporothrix* spp. and species-specific PCR identified the isolate as *S. brasiliensis* [23]. The isolate exhibited a wild-type profile to itraconazole and amphotericin B, with a MIC of 0.125 µg/mL and 4 µg/mL, respectively [24,25].

One month later, she returned reporting discontinuation of both itraconazole and ART, presenting worsening of the multifocal cutaneous lesions, as well as axillary and cervical lymphadenopathy. Oral itraconazole (200 mg/day) and ART were therefore reinitiated. After 15 days of treatment, clinical re-evaluation demonstrated partial improvement, characterized by crust formation and healing of some lesions. Nevertheless, a draining lesion on the right buttock, persistent axillary lymphadenopathy, and polyarthralgia were still observed. Later that month, the patient presented again to the SRS/HU-FURG/Ebserh with painful skin lesions, joint pain, and edema of the feet. New crusted lesions had developed on her face, trunk, and limbs, accompanied by worsening of the right eye lesion (Figure 1B).

At that time the patient was hospitalized to initiate the therapy with intravenous amphotericin B deoxycholate (50 mg/day; Cristália, Itapira, São Paulo, Brazil) for eight days. ART was reintroduced using the same regimen, and following completion of amphotericin B therapy, oral itraconazole was prescribed at a PO dose of 400 mg/day. After 39 days of itraconazole therapy, the patient exhibited complete resolutions of the lesions and a satisfactory general clinical condition. Although a follow-up visit was scheduled for the following week, she did not return for further evaluation.

In January 2026, the patient returned to the SRS/HU-FURG/Ebserh presenting ulcerative and crusted lesions on her nose, arms, and left leg (Figure 1C). She reported irregular adherence to ART and itraconazole treatment during the period without outpatient follow-up. At this time, the HIV viral load was 302,000 copies/mL and the CD4+ T-cell count was low again (114 cells/mm^3^). Swab samples of lesion secretions were collected for mycological analysis. Mycological culture confirmed the isolation of *Sporothrix* spp., and the isolate was identified as *S. brasiliensis* by species-specific PCR [23]. *In vitro* susceptibility testing reaffirmed its wild-type profile to itraconazole and amphotericin B, with a MIC of 0.25 µg/mL and 1 µg/mL, respectively [24,25].

At that time ART was reintroduced using TDF (300 mg/day), 3TC (300 mg/day), and DTG (50 mg/day). However, the patient declined hospitalization for amphotericin B administration; therefore, a treatment regimen consisting of oral itraconazole at 400 mg/day (200 mg every 12 h) was prescribed. In April 2026, the patient returned to the SRS/HU-FURG/Ebserh, presenting worsening of the nose, arms, and legs lesions, as well as the appearance of new lesions on the lips, feet, arms, and legs. Despite clinical progression, she again refused hospitalization, and itraconazole at 400 mg/day (200 mg every 12 h) was maintained. The patient did not return for subsequent outpatient follow-up visits.

### 3.2. Case 2

**Figure 2:**
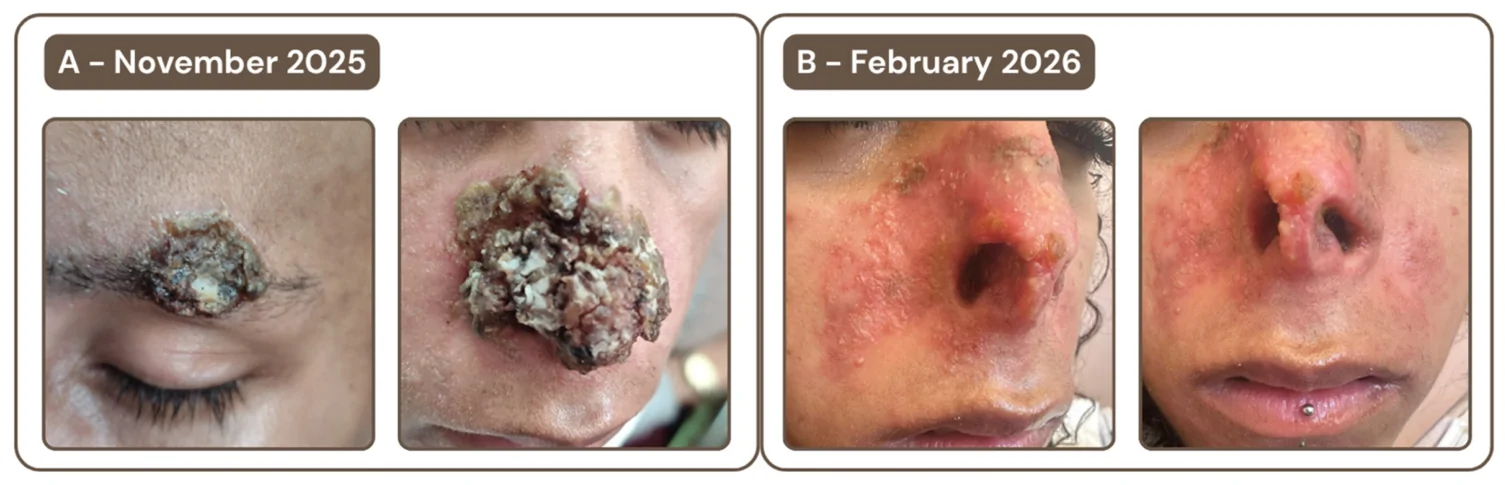
A 31-year-old transgender woman who reported contact with a stray cat two to four months before the onset of extensive mucocutaneous sporotrichosis, which subsequently progressed to multifocal disease. The diagnosis of HIV infection was established concomitantly with the diagnosis of sporotrichosis. (**A**) Initial clinical presentation of sporotrichosis caused by *Sporothrix brasiliensis* in November 2025. (**B**) Clinical progression in February 2026 after 26 days of treatment with intravenous liposomal amphotericin B (3 mg/kg/day) as a loading regimen.

On 20 November 2025, a 31-year-old transgender woman who self-identified as belonging to the Brazilian *parda* racial/ethnic category sought care at the SRS/HU-FURG/Ebserh, Rio Grande, RS, Brazil. The patient presented with crusted lesions involving the nasal mucosa, nasal skin, and left eyebrow (Figure 2A). She reported contact with a stray cat two to four months before presentation, during which the animal sneezed on her face. During the outpatient evaluation, a rapid HIV test yielded a reactive result, and samples of nasal lesion secretions were collected for mycological examination. Laboratory evaluation revealed an HIV viral load of 522,000 copies/mL and a CD4+ T-cell count of 55 cells/mm^3^.

Mycological culture resulted in the isolation of *Sporothrix* spp., and direct mycological examination revealed yeast cells suggestive of this fungus. The species was identified as *S. brasiliensis* by species-specific PCR [23]. The isolate exhibited a wild-type profile for *in vitro* susceptibility to itraconazole, with a MIC of 0.125 µg/mL, and a wild-type profile for amphotericin B susceptibility, with a MIC of 0.5 µg/mL [24,25]. Considering the risk of IRIS, oral itraconazole was initiated at a dose of 200 mg/day (Geolab^®^, Anápolis, Goiás, Brazil), and antiretroviral therapy was postponed for seven days. After this period, the patient initiated ART with TDF (300 mg/day), 3TC (300 mg/day), and DTG (50 mg/day), and remained under outpatient follow-up.

In January 2026, the patient was hospitalized at HU-FURG/Ebserh after reporting treatment abandonment, showing progression for a severe nasal lesion, characterized by partial destruction of the nasal tissue, increased purulent discharge and focal pain, as well as the appearance of new ulcerative lesions on the left forearm and right leg. The patient remained hospitalized for 26 days and received a loading regimen of intravenous liposomal amphotericin B (3 mg/kg/day; AmBisome^®^, Gilead Sciences, Foster, CA, USA), in addition to oral antiretroviral therapy consisting of tenofovir alafenamide (TAF; 25 mg/day), 3TC (300 mg/day), and DTG (50 mg/day). By February 2026 the patient showed marked clinical improvement, including detachment of crusts from the facial and forearm lesions, absence of new lesions, and satisfactory wound healing (Figure 2B). Subsequently, treatment was switched to oral itraconazole at a dose of 400 mg/day (200 mg every 12 h). The patient tolerated the treatment well, presenting only mild hepatic alterations, and was discharged for outpatient follow-up at the SRS/HU-FURG/Ebserh. In May 2026, she returned for follow-up, showing significant clinical improvement, although a residual nasal deformity remained, while the other lesions were in the healing process.

## 4. Discussion

We report two cases of severe mucocutaneous sporotrichosis in PLHIV, both evolving to multifocal cutaneous lesions, highlighting the severity that this mycosis can reach in immunosuppressed individuals, even resulting in permanent destruction and scarring sequelae. Extensive mucocutaneous involvement leading to fibrosis and deformation of mucosal and cartilaginous structures is uncommon among sporotrichosis reports, even among PLHIV [26,27]. To our knowledge, only one similar case has been reported in another Brazilian hyperendemic area for sporotrichosis, Rio de Janeiro state. Eyer-Silva et al. [28] described *S. brasiliensis* sporotrichosis in a PLHIV who developed severe mucosal involvement, including a palatal ulcer, uvular destruction, and nasal septum perforation. Such deforming sequelae are more commonly associated with other endemic mycoses, particularly paracoccidioidomycosis [29].

Therefore, our report expands the spectrum of severe manifestations associated with sporotrichosis and reinforces that, in immunosuppressed patients, this disease may result in destructive and permanently disfiguring lesions similar to those observed in other systemic mycoses. In addition, these cases add to previous reports from RS, another Brazilian hyperendemic region for sporotrichosis since the 1990s, where only a few severe and multifocal cases have been documented despite more than three decades of hyperendemicity [14,18,21,22]. Previously reported non-cutaneous forms of sporotrichosis in this region consisted mainly of ocular manifestations or hypersensitivity reactions, which are generally associated with a more favorable prognosis [8,9]. These findings may suggest a change in the local epidemiology of sporotrichosis in the region; however, the number of cases described here are insufficient to sustain this hypothesis, thus prospective studies are needed to confirm it.

Reviewing the frequency of cutaneous multifocal sporotrichosis associated with *S. brasiliensis*, we identified 22 articles using the descriptors described in the Methods section. Among these, only six described the occurrence of cutaneous multifocal sporotrichosis, encompassing 87 cases of this manifestation [16,21,30,31,32,33]. However, among the case series, the reported frequency of cutaneous multifocal sporotrichosis ranged from 0.5% to 8.9% [16,30,33]. The highest frequency (8.9%; 81/911) was reported in a study involving older patients; however, all case series reported comorbidities for the overall study population, without differentiating them according to the clinical manifestation of sporotrichosis [16,30,33]. Therefore, the available data do not allow us to determine whether the reported comorbidities are specifically associated with cutaneous multifocal sporotrichosis. Only two case reports specifically described comorbidities in patients with this manifestation: one involving HIV infection and the other diabetes mellitus [21,31]. In the systematic review including only PLHIV, multifocal cutaneous sporotrichosis accounted for 16% of cases (8/51), whereas multifocal extracutaneous disease was the most prevalent manifestation (78%; 40/51). Among patients with multifocal extracutaneous sporotrichosis, multiple organ systems were affected, including the central nervous system (*n* = 21), respiratory system (upper and lower airways; *n* = 10), visual system (*n* = 9), and skeletal system (*n* = 9) [17]. Given all the available data from the general population and PLHIV, the limited evidence on cutaneous multifocal sporotrichosis caused by *S. brasiliensis* highlights the need for further studies to investigate its association with underlying conditions, lesion severity, treatment, and clinical outcomes.

In our cases, the progression to mucocutaneous multifocal sporotrichosis was directly associated with HIV infection and low CD4+ T-cell counts. Both cases were also marked by discontinuation of ART, particularly the first patient, who did not achieve clinical cure of sporotrichosis after approximately two years of disease progression. This patient presented social problems, including anomie and substance abuse, which influenced her decision to decline the most intensive treatment (amphotericin B), contributing to a worse prognosis and disease dissemination [34].

The second patient initially presented with extensive but non-multifocal lesions, which progressed to cartilage and mucosal destruction. The severity of this clinical presentation prompted investigation for HIV infection, resulting in the concomitant diagnosis of HIV infection and sporotrichosis. We highlight that itraconazole therapy was initiated in the second patient immediately after the diagnosis of sporotrichosis, whereas antiretroviral therapy was delayed for one week to avoid IRIS, as previously reported in our region at the same center [21]. Her subsequent discontinuation of ART likely contributed to the dissemination of *S. brasiliensis* infection and the need for treatment with intravenous amphotericin B. The patient showed a favorable clinical outcome during follow-up, with significant improvement of the lesions. However, extensive cartilage destruction resulted in permanent sequelae associated with sporotrichosis, contributing to morbidity and potentially affecting the patient’s long-term quality of life. These findings reinforce the importance of investigating underlying immunological impairment, particularly HIV infection, in patients presenting with severe cutaneous sporotrichosis, as well as ensuring adherence to ART to prevent disease progression and complications [34,35].

Although our patients had advanced HIV infection and severe multifocal cutaneous disease, neither developed visceral sporotrichosis, in contrast to reports from other hyperendemic areas [6,8,10,32,36,37,38]. This difference may be associated with the genotypes of *S. brasiliensis* circulating in RS, which appear to differ from those reported in Rio de Janeiro and other endemic states [39,40]. Previous studies have suggested that the clinical profile of sporotrichosis in RS is more frequently characterized by forms associated with a better prognosis, including cutaneous disease (even multifocal forms), ocular manifestations, and hypersensitivity reactions, rather than systemic involvement [8,9,14,18,19]. However, further studies are needed to evaluate the relationship between *S. brasiliensis* genotypes and clinical manifestations across different hyperendemic regions.

Another noteworthy finding of our study pertains to the concern for emergence of decreased antifungal susceptibility in *S. brasiliensis*. Treatment interruption during prolonged antifungal therapy is recognized as an important factor contributing to the development of azole resistance, as repeated exposure to suboptimal drug concentrations may exert selective pressure and promote the emergence of resistant strains [41]. However, despite the chronic course of infection and intermittent treatment observed in our patients, isolates recovered during their courses remained wild-type with respect to itraconazole and amphotericin B susceptibility [25,40]. We emphasize the importance of continued surveillance to establish potential correlations between clinical outcomes and *in vitro* susceptibility profiles [3].

Our study has some limitations that should be acknowledged. First, as this was a case series, the findings should be interpreted within the context of the cases described and may not be generalizable to all cases of sporotrichosis associated with HIV infection or to all cases occurring in our hyperendemic region. Prospective epidemiological studies are needed to evaluate whether the apparent increase in severe cases represents a true epidemiological trend and to better characterize the burden of sporotrichosis among PLHIV in our region. Furthermore, the loss to follow-up of patient 1 prevented the assessment of the final clinical outcome, thereby limiting definitive conclusions regarding the case. Despite these limitations, the cases provide valuable clinical and epidemiological information regarding severe sporotrichosis in PLHIV from a hyperendemic region, contributing to the recognition of factors associated with unfavorable outcomes and permanent sequelae.

## 5. Conclusions

In conclusion, these cases highlight the potential severity of *S. brasiliensis* mucocutaneous infection in PLHIV, particularly in the context of advanced immunosuppression and inadequate adherence to antiretroviral and antifungal therapies. The progression to multifocal lesions, extensive tissue destruction, and permanent sequelae observed reinforces the need for early HIV investigation in severe presentations of sporotrichosis, as well as prompt diagnosis and appropriate clinical management. Furthermore, the occurrence of these cases in a hyperendemic region where severe forms have been rarely reported underscores the importance of continued epidemiological surveillance and clinical awareness to improve patient outcomes and better understand the evolving epidemiology of sporotrichosis.

## Data Availability

No additional data were generated in this study; all data are available in the article.
